# A Bioelectrically Enabled Smart Bandage for Accelerated Wound Healing and Predictive Monitoring

**DOI:** 10.3390/medicina61060965

**Published:** 2025-05-23

**Authors:** Ahmad F. Turki, Aziza R. Alrafiah

**Affiliations:** 1 Electrical and Computer Engineering Department, Faculty of Engineering, King Abdulaziz University, Jeddah 21589, Saudi Arabia; 2 Center of Excellence in Intelligent Engineering Systems (CEIES), King Abdul Aziz University, Jeddah 21589, Saudi Arabia; 3 Department of Medical Laboratory Sciences, Faculty of Applied Medical Sciences, King Abdulaziz University, Jeddah 21589, Saudi Arabia; aalrafiah@kau.edu.sa

**Keywords:** wearable technologies, smart bandage, wound healing, electric field therapy, advanced wound care, machine learning

## Abstract

*Background and Objectives:* Chronic wounds pose a significant healthcare burden due to their prolonged healing times and susceptibility to infection. Electric field (EF)-enabled smart bandages offer a promising solution by combining therapeutic stimulation with real-time physiological monitoring. *Materials and Methods:* This study assessed a smart bandage integrating spiral stainless steel electrodes delivering a 200 millivolts per millimeter (mV/mm) EF for 5 h daily over 14 days to full-thickness excisional wounds in 100 Sprague–Dawley rats. Vital signs including heart rate (BPM), oxygen saturation (SpO_2_), and temperature were monitored continuously. Machine learning models were trained on these data to predict wound healing status. *Results:* By Day 7, EF-treated wounds demonstrated significantly faster healing, achieving an average wound closure rate of 82.0% ± 2.1% compared to 70.75% ± 2.3% in the control group (*p* < 0.05). By Day 14, wounds in the experimental group had significantly reduced to 0.01 ± 0.005 cm^2^, while the control group retained a wound size of 0.24 ± 0.03 cm^2^ (*p* < 0.05). Histological analysis revealed enhanced neovascularization, collagen alignment, and epithelial regeneration in the EF group. Physiological data showed no systemic inflammatory response. Predictive modeling using XGBoost and Random Forest achieved >98% accuracy, with SHAP (SHapley Additive exPlanations) analysis identifying EF exposure and treatment duration as key predictors. *Conclusions:* The findings demonstrate that EF-based smart bandages significantly enhance wound healing and enable highly accurate prediction of outcomes through machine learning models. This bioelectronic approach holds strong potential for clinical translation.

## 1. Introduction

The skin, the body’s largest organ, plays a critical role in protecting against environmental pathogens, regulating body temperature, and preventing dehydration [1]. A wound, defined as a disruption in the structural integrity of the skin, can result from trauma, illness, surgical intervention, or prolonged pressure [2]. While acute wounds typically follow a predictable healing trajectory, chronic wounds such as diabetic foot ulcers, venous leg ulcers, and pressure ulcers exhibit prolonged healing durations, persistent inflammation, and disrupted extracellular matrix (ECM) remodeling [3,4,5,6]. These wounds fail to close within the expected timeframe, typically remaining open for more than 30 days, and often result in complications such as necrosis, infection, and, in severe cases, limb amputation [6].

Globally, approximately 2% of the population is affected by chronic wounds, contributing to substantial healthcare costs and patient morbidity [7]. In Saudi Arabia, the burden of chronic wounds is intensified by a high prevalence of diabetes and cardiovascular conditions [8]. In 2022, over 4.27 million adults in Saudi Arabia 17.7% of the population were reported to have diabetes, placing them at elevated risk for developing diabetic foot ulcers [8]. Up to 25% of diabetic individuals may experience a foot ulcer during their lifetime [8]. Moreover, additional chronic conditions such as heart disease and hypertension present in approximately 24.2% of individuals in some regions further hinder wound healing [9]. These realities highlight an urgent need for targeted and technology-driven wound management strategies in the local healthcare context.

In the United States alone, more than 6.7 million individuals suffer from chronic non-healing wounds, resulting in annual healthcare expenditures exceeding USD 25 billion [10].

Wound healing is a multifaceted biological process involving tissue regeneration, angiogenesis, immune modulation, and extracellular matrix remodeling [11]. Conventional wound dressings primarily serve as protective barriers, offering little active support for the healing process [12].

Despite significant advancements in the development of new dressing materials and clinical protocols, current wound care practices remain largely reactive [13]. Most treatments rely heavily on visual assessment and the clinical experience of the healthcare provider [14]. While therapies like skin grafts, negative pressure wound therapy, and skin substitutes can aid healing, they are often invasive, resource-intensive, and limited in their ability to adapt to a wound’s dynamic condition [15,16]. Moreover, frequent use of antibiotics to manage infection risks has led to rising concerns over antimicrobial resistance, compounding the complexity of chronic wound care [17].

To address these limitations, researchers have increasingly focused on smart bandage technologies that integrate therapeutic action with real-time monitoring capabilities [18].

Traditional wound dressings such as gauze and cotton offer passive protection but often adhere to the wound site, leading to secondary trauma and dehydration [19]. In contrast, advanced conductive dressings based on electroactive biomaterials have shown promising outcomes by actively modulating cellular behavior through electrical stimulation [19]. Our spiral stainless steel electrode is designed to harness these benefits with additional structural advantages: (1) the spiral configuration enhances surface conformity and ensures uniform current distribution across the wound bed, reducing hotspots and associated tissue irritation; (2) stainless steel offers excellent conductivity, mechanical strength, biocompatibility, and corrosion resistance, which are essential for stable and reusable bioelectronic applications; and (3) unlike many conductive polymers and nanomaterials that require complex synthesis or suffer from in vivo degradation issues, stainless steel provides long-term electrochemical stability under physiological conditions [19]. These features collectively position the spiral stainless steel electrode as a superior platform for bioelectrically enhanced wound healing, consistent with recent trends in conductive biomaterials for tissue regeneration [19].

Recent advances in biomedical engineering have paved the way for smart bandage technologies that integrate electric field (EF) stimulation and biometric monitoring, offering a transformative approach to chronic wound care [20].

Endogenous electric fields are naturally generated at injury sites and play a critical role in guiding cell migration, promoting proliferation, and coordinating tissue repair [21,22,23,24]. External EF application has been shown to enhance these biological signals, accelerating re-epithelialization, angiogenesis, and ECM remodeling [25].

Externally applied EFs have been shown to amplify these effects by promoting fibroblast and keratinocyte migration, enhancing epithelialization, increasing neovascularization, and aligning collagen fibers, leading to improved tissue integrity and reduced scarring [20]. Several preclinical studies have confirmed that EF-treated tissues exhibit faster wound closure, denser microvascular networks, and superior collagen organization, suggesting that bioelectric stimulation holds therapeutic potential beyond conventional treatment modalities [21,22,23,24,25,26,27,28,29].

Mechanistically, EF stimulation activates key cellular pathways such as phosphoinositide 3-kinase/protein kinase B (PI3K/Akt), directing the migration of keratinocytes, fibroblasts, and endothelial cells toward the wound site [30]. It also enhances vascular endothelial growth factor (VEGF) expression, promoting neovascularization essential for delivering oxygen and nutrients to regenerating tissue [31]. Moreover, electric fields have been linked to increased collagen synthesis and alignment, which contributes to stronger skin regeneration and reduced scarring [32].

Alongside EF stimulation, the use of biomarkers derived from wearable biosensors such as heart rate (BPM), oxygen saturation (SpO_2_), and temperature has emerged as a promising method for monitoring wound status in real time [33]. These physiological signals reflect underlying systemic conditions and local tissue responses, providing a dynamic profile of wound progression or stagnation [33]. Unlike static clinical evaluations, continuous biometric monitoring enables personalized assessment and early detection of complications such as infection, delayed healing, or circulatory dysfunction [33].

Emerging technologies now combine these capabilities into smart bandage systems that not only apply therapeutic electric stimulation but also gather continuous health data. When coupled with machine learning (ML) techniques, these data can be leveraged to predict wound healing outcomes, creating a feedback-enabled platform for adaptive wound care [34]. Recent efforts, including work by Jiang et al. [35] and Hossain et al. [36], have shown that smart bandages integrated with biosensors and predictive algorithms can meaningfully improve healing outcomes and reduce clinician dependency on visual inspection alone.

Despite this promise, widespread clinical adoption of such devices remains limited by technical challenges, including sensor reliability, power management, and the lack of validated ML frameworks for wound prediction [12,37]. Nevertheless, the integration of EF therapy with predictive modeling based on physiological biomarkers represents an exciting direction in regenerative medicine, particularly for chronic wound care.

This study aims to evaluate the efficacy of a spiral stainless steel electrode-based smart bandage system delivering electric field stimulation in accelerating wound healing, improving tissue strength, collagen alignment, and reducing scar formation. In parallel, we investigate the potential of using biometric markers (heart rate, SpO_2_, and temperature) for real-time prediction of wound healing status through supervised machine learning models. By combining therapeutic intervention with intelligent monitoring and data-driven prediction, this research seeks to advance the development of next-generation smart wound care solutions.

## 2. Materials and Methods

### 2.1. Smart Bandage Design

The smart bandage system is composed of several integral components that work together to facilitate both electric field (EF) generation and biometric monitoring. The EF was generated using stainless steel alloy wire electrodes, with a 200 mV/mm strength applied at a 1 cm distance between electrodes. A 2 V regulated output was used, controlled by a step-down voltage regulator to maintain consistency and safety. The electric field strength (*E*) in the system is determined using [38]

(1) $$E=\frac{\;V}{d}$$

where *V* = 2 volts and *d* = 0.01 m (1 cm), resulting in an EF strength of 200 mV/mm, which falls within the optimal range for stimulating cell migration and wound healing.

The electric field strength (*E*) in the system was calculated using Equation (1) and set to 200 mV/mm, a value selected based on previous studies demonstrating its effectiveness in promoting directed cell migration, enhancing keratinocyte and fibroblast activity, and accelerating tissue regeneration. This field strength lies within the optimal therapeutic range (100–300 mV/mm) reported to facilitate wound healing processes through modulation of endogenous bioelectric signaling [20,21,39].

A 420 mAh Lithium Polymer (LiPo) battery provided the necessary power to sustain these functions efficiently. To regulate the voltage distribution, the step-down regulator ensures a stable 2 V output essential for generating the electric field, while a step-up regulator increases the voltage to 5.5 V to adequately power the NodeMCU ESP8266 (Wi-Fi microcontroller unit) (Espressif Systems, Shanghai, China). This microcontroller plays a pivotal role in controlling system operations, processing data from sensors, and wirelessly transmitting information to a cloud-based monitoring platform. For real-time health monitoring, the MAX30102 sensor (Analog Devices, Wilmington, MA, USA) continuously tracks vital parameters, including heart rate (BPM), oxygen saturation (SpO_2_), and temperature (°C).

The used electrodes were strategically placed to enhance cellular migration and tissue regeneration, expediting the healing process. The electrodes were integrated into a conductive hydrogel layer positioned to maintain stable contact around the wound periphery, allowing uniform EF delivery without disrupting crust formation.

**Figure 1:**
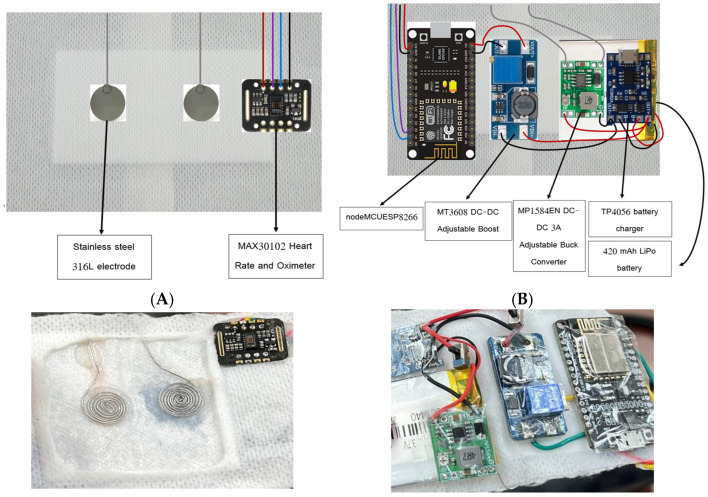
Photographic illustration of the fabricated smart bandage system. (**A**) Front view showing the placement of spiral stainless steel (316L) electrodes and the MAX30102 sensor for heart rate and oxygen saturation monitoring. (**B**) Rear view displaying key electronic components including a NodeMCU ESP8266, MT3608 DC–DC boost converter, MP1584EN buck converter, TP4056 battery charger, and 420 mAh LiPo battery.

Figure 1 illustrates the electronic architecture of the smart bandage system, detailing power regulation, control, and sensor integration.

**Figure 2:**
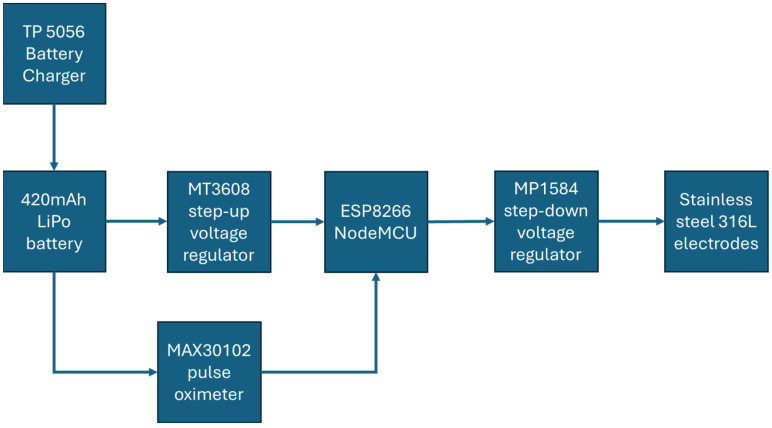
Block diagram of the designed smart bandage system.

Figure 2 presents the block diagram of the system, summarizing the operational flow and component relationships within the smart bandage.

The migration velocity ($v_{c}$) of cells under an applied EF is determined by [39]

(2) $$v_{c}=\mu E$$

where *μ* is the cell mobility coefficient and *E* is the electric field strength. This equation describes galvanotaxis (electrotaxis), a phenomenon where cells migrate directionally in response to an electric field [39]. The applied EF generates a force on charged cellular components, particularly influencing keratinocytes, fibroblasts, and endothelial cells, guiding their movement toward the wound site [39]. By precisely controlling the EF strength, the system can accelerate wound closure by enhancing cellular motility, extracellular matrix remodeling, and neovascularization. Experimental studies indicate that fibroblasts and keratinocytes respond to electric fields between 200 and 300 mV/mm, contributing to enhanced epithelialization and dermal reconstruction [40].

Additionally, the force (*F*) exerted on a charged particle in the electric field is given by Coulomb’s Law:

(3) F=qE

where *q* represents the net charge of a cell membrane, and *E* is the applied electric field strength [41]. This equation explains how keratinocytes and fibroblasts experience directional movement under the influence of EF [41]. The applied force stimulates intracellular signaling cascades, activating cytoskeletal rearrangement and cellular polarization, both of which are critical for effective wound closure [40].

Furthermore, the distribution of the electric field within tissue follows Laplace’s equation for steady-state conditions:

(4) $$\nabla^{2}V=0$$

where $\nabla^{2}$ is the Laplacian operator, representing the sum of the second partial derivatives of the electric potential (V) in three-dimensional space [41]. This equation describes how the electric potential propagates throughout the wound environment, ensuring a uniform and stable EF distribution across the tissue [41]. Since biological tissues exhibit conductivity, the EF influences cell polarization, membrane depolarization, and tissue remodeling—all of which are critical factors in regenerative healing [41]. The uniform electric field distribution ensures consistent stimulation of migrating cells, optimizing keratinocyte and fibroblast alignment, and promoting efficient wound contraction [41].

Controlled and localized current distribution plays a pivotal role in modulating cellular behavior, primarily by enhancing ion channel activity. This bioelectrical stimulation facilitates key regenerative processes, including fibroblast proliferation, ECM remodeling, and tissue regeneration. The EF induced current density (*J*) in this study was calculated using the following formula [42]:

(5) J=σE

where *σ* is the skin conductivity (≈0.2 S/m) and *E* is the applied field (200 mV/mm).

### 2.2. Study Samples

This study involved 100 healthy adult male Sprague–Dawley rats subjected to controlled wound healing protocols. The animals were randomly divided into two equal groups: the blank group and the apparatus group. The experimental group received treatment with the smart bandage (EF rat), while the control group healed naturally without electric field stimulation (C rat).

This study adhered to ethical standards, with approval from the Institutional Review Board (IRB) of the Center of Excellence in Intelligent Engineering Systems at King Abdulaziz University (approval number: 27-CEIES-Bioeth-2024).

### 2.3. Experimental Protocol

Full-thickness excisional wounds measuring 2.0 cm in length, width, and depth were surgically created on the dorsal skin of each rat under sterile conditions. Prior to incision, the animals were anesthetized via intraperitoneal injection of xylazine (10 mg/kg) and ketamine (50 mg/kg). The dorsal neck region was shaved and sterilized with 70% alcohol, and a 1 cm midline incision was made through the skin and subcutaneous tissue.

**Figure 3:**
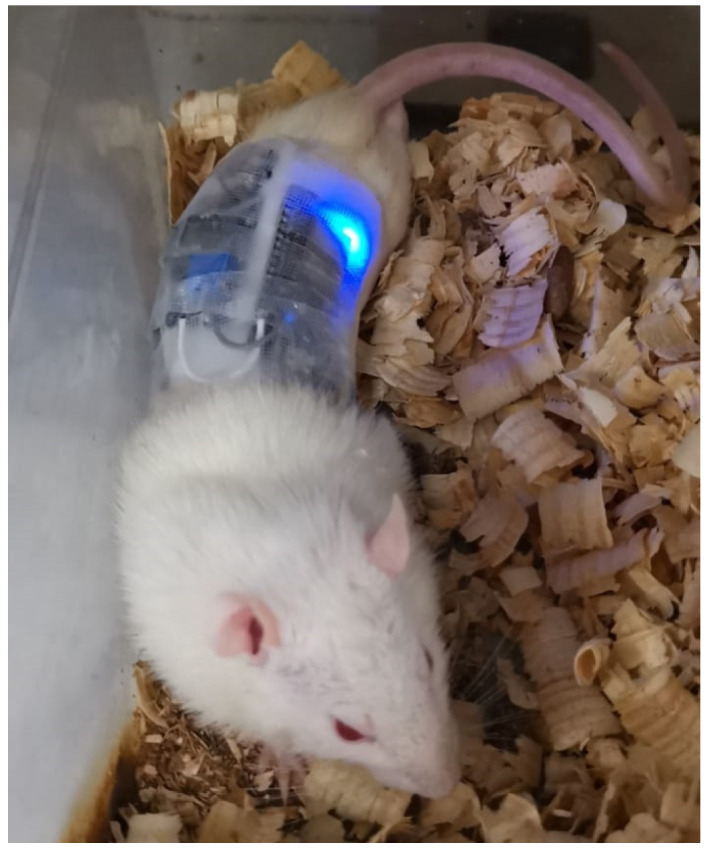
Image of mice freely moving while wearing wireless smart bandages. Blue LED light indicates active system operation.

Immediately following wound induction, treatment protocols were initiated. In the experimental group, a smart bandage system was applied directly over the gauze dressing to ensure optimal electrode contact with the wound site. This system delivered a controlled electric field (EF) stimulation by applying a 2 V potential across stainless steel electrodes, generating a localized EF of 200 mV/mm. The EF device operated continuously for 5 h per day over a 14-day period, providing a consistent therapeutic dose. Simultaneously, the smart bandage continuously monitored and recorded key physiological parameters—heart rate (BPM), oxygen saturation (SpO_2_), and body temperature (°C)—which were transmitted to a cloud-based platform for real-time tracking and analysis. The wireless design allowed for unrestricted animal movement throughout the treatment period, as demonstrated in Figure 3. 

In the control group, wounds were covered with standard gauze dressings without EF stimulation, though the same biometric monitoring system was used to collect physiological data for comparative analysis. All rats were housed in a controlled environment to ensure stable monitoring conditions throughout this study.

Wound healing progression was assessed through standardized high-resolution imaging conducted hourly during each 5-h treatment session across the 14-day period. A fixed camera setup ensured consistency in angle, distance, and lighting, with a metric scale included for calibration. Macroscopic evaluation was conducted at three key time points—Days 3, 7, and 14—while wound area was quantified using MATLAB R2022b (MathWorks, Natick, MA, USA) by tracing wound margins and calculating area changes. Wound closure percentages were computed based on these measurements to provide an objective and non-invasive metric for healing dynamics.

### 2.4. Histological Analysis

Histological samples were collected on designated days (Days 3, 7, and 12) to investigate tissue regeneration at the microscopic level. Histological staining was performed using hematoxylin and eosin (H&E) and Masson’s trichrome techniques.

For H&E staining, skin sections fixed in 10% neutral-buffered formalin were embedded in paraffin, sectioned at 5 μm, and then processed through standard histological protocols. Slides were deparaffinized in xylene, rehydrated through graded ethanol, and stained with hematoxylin to visualize cell nuclei. Following a brief rinse in tap water, sections were counterstained with eosin to highlight cytoplasmic and extracellular matrix components. After dehydration and clearing, the slides were mounted for microscopic analysis.

Masson’s trichrome staining followed a similar preparation, with paraffin-embedded sections undergoing deparaffinization, rehydration, and sequential staining. Nuclei were stained using Weigert’s iron hematoxylin; this was followed by Biebrich scarlet–acid fuchsin to stain cytoplasm and muscle fibers and aniline blue to selectively stain collagen fibers. This differential staining allowed for clear visualization of dermal connective tissue organization and collagen remodeling.

### 2.5. Wound Healing Assessment

Wound healing progression was assessed using standardized digital imaging conducted hourly each day during the 5-h treatment sessions over a 14-day period. High-resolution photographs were consistently captured at a fixed angle and distance under controlled lighting conditions to ensure reproducibility. A metric scale was included in each image frame to allow for precise calibration of wound measurements. Image analysis was carried out using MATLAB R2022b software (MathWorks, Natick, MA, USA), which was utilized to trace wound margins and calculate wound area. This approach enabled accurate quantification of wound dimensions at each time point. The resulting measurements were then used to compute wound closure percentages over time, providing a non-invasive, reproducible, and objective method for monitoring healing dynamics. The wound closure rate was determined by measuring the wound area using an image analysis program. The percentage of wound closure was calculated using the following formula:

*Wound closure (%) = [*1 *− (open wound area/initial wound area)] ×* 100 (6)

This quantitative method provided an objective evaluation of the effectiveness of the smart bandage in accelerating wound healing.

### 2.6. Machine Learning-Based Wound Classification

To explore the predictive potential of physiological biomarkers in evaluating wound healing status, a machine learning (ML) approach was integrated into the experimental framework. The primary objective was to classify wound condition as either “healed” or “not healed” based on biometric parameters collected by the smart bandage system. The classification labels were derived from the percentage of wound closure, where wounds achieving ≥80% closure were classified as “healed” and those below this threshold as “not healed”.

A total of approximately 7000 data points were gathered from 100 Sprague–Dawley rats, with data recorded over 14 days and 5 h per day. For each animal and time point, the smart bandage system captured real-time biometric features including healing duration (day number), electric field treatment status (treated or control), heart rate (BPM), oxygen saturation (SpO_2_), and temperature (°C).

These features were used as input variables, while the binary wound condition served as the target variable. The dataset was preprocessed to handle missing values, ensure consistency in data formatting, and normalize feature values where necessary.

Four supervised ML algorithms were implemented Logistic Regression, Random Forest Classifier, Support Vector Machine (SVM), and Extreme Gradient Boosting (XGBoost)

The dataset was randomly split into training (80%) and testing (20%) subsets using stratified sampling to maintain class balance. Each model was trained using the training subset and evaluated on the testing set using the following performance metrics:

Accuracy: This metric assesses the overall correctness of the model by calculating the ratio of correctly classified instances (both true positives and true negatives) to the total number of instances [42]. It provides a general measure of the model’s effectiveness in making accurate predictions across all classes [42].

(7) $$Accuracy=\frac{\left(TP+TN\right)}{\left(TP+TN+FP+FN\right)}\times 100$$

Precision: This metric emphasizes the accuracy of the model’s positive predictions by calculating the proportion of true positives among all instances predicted as positive [43]. Precision reflects the model’s ability to avoid false positives, indicating how reliable its positive predictions are [42].

(8) $$Precision=\frac{TP}{\left(TP+FP\right)}\times 100$$

Recall (or sensitivity): This metric evaluates the model’s ability to identify all relevant instances in the dataset [43]. It is calculated as the proportion of true positives out of all actual positive instances, indicating how effectively the model captures the true positives and minimizes false negatives [42].

(9) $$Recall=\frac{TP}{\left(TP+FN\right)}\times 100\;$$

where, for Equations (7)–(9), TP is true positive, TN is true negative, FP is false positive, and FN is false negative.

F1 Score: This metric provides a balanced evaluation of a model’s performance across individual classes, offering a more nuanced assessment than overall accuracy alone [43]. The F1 score combines precision and recall, calculating their harmonic mean to deliver a single metric that accounts for both false positives and false negatives [42]. This makes it particularly useful for models where balancing precision and recall is essential, providing a comprehensive measure of the model’s predictive ability [42].

(10) $$F1\;Score=2\times \frac{Precision\;x\;Recall}{\left(Precision+Recall\right)}\times 100$$

Area Under the Receiver Operating Characteristic Curve (AUC): The AUC quantifies the overall ability of a classification model to distinguish between classes by measuring the area under the receiver operating characteristic (ROC) Curve, which plots the true positive rate (sensitivity) against the false positive rate (1-specificity) [42]. A higher AUC indicates better model performance across various classification thresholds [43]. Mathematically, it can be expressed as follows:

(11) $$AUC=\int_{0}^{1}ROC\;curve\left(TP\right)\;d\left(FP\right)\times 100$$

To interpret feature contributions, SHAP (SHapley Additive exPlanations) analysis was conducted on the top-performing model, allowing for quantification of the influence of each feature on the model’s output [42]. This provided insights into the physiological and temporal parameters most critical for distinguishing between healed and non-healed wounds.

(12) $$\phi \left(f\right)=\phi_{0}+\sum_{j=1}^{M}\frac{f\left(x_{i}\right)-E\left[f\left(x_{i}\right)\right]}{M}$$

where

*ϕ*(*f*) represents the SHAP values for a particular feature;

*ϕ*_0_ is the expected value of the model’s prediction;

*M* is the total number of features;

*f*(*x_i_*) is the model’s prediction when feature *i* is included;

*E*[*f*(*x_i_*)] is the expected prediction when feature *i* is excluded.

## 3. Results

### 3.1. Wound Healing Progress

**Figure 4:**
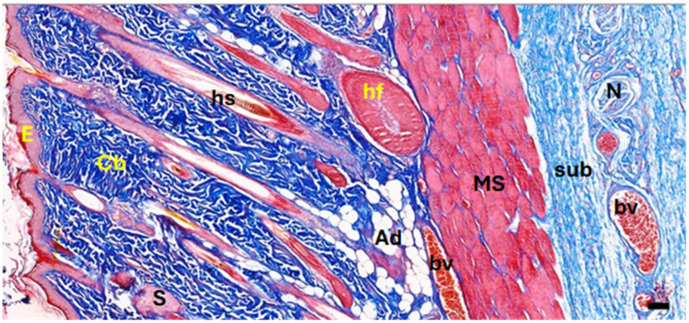
Photomicrograph of normal back skin of one of the experimental rats showing the cutaneous layers and their contents. Epidermis (E), collagenous bundles (Cb), hair shaft (hs), hair follicle (hf), sebaceous gland (S), adipose tissue (Ad), blood vessels (bv), nerves (N), cutaneous muscle (MS), and subcutaneous tissue (sub). Magnification: ×40. Stain: MS. Bar: 50 micrometers (µm).

In the current study, surgical wounds were created in the back regions of experimental rats, leading to the removal of full skin layers. Normal skin contains the epidermis, dermis, collagenous bundles, hair follicles, adipose tissue, blood vessels, nerves, and cutaneous muscle layers (Figure 4). After healing, the dermis in both the control and EF-treated groups showed significant modifications. The EF-treated group exhibited increased neovascularization in the subepithelial layer and a higher density of fibroblastic nuclei, suggesting active tissue regeneration, whereas the control group displayed a dominance of collagenous fibers with fewer cellular elements.

The wound healing process was meticulously monitored throughout this study, revealing consistent and pronounced differences between the EF-treated and control groups. At Day 0, both groups began with identical full-thickness excisional wounds measuring 2.0 cm × 2.0 cm. By Day 10, the EF-treated group showed substantial improvement, with average wound dimensions reduced to 0.6 cm in length and 0.5 cm in width, while the control group maintained larger dimensions of 0.9 cm in length and 0.6 cm in width. Continued progression was evident by Day 12, as the EF group wounds were further reduced to 0.3 cm × 0.3 cm compared to 0.75 cm × 0.45 cm in the control group. Visual comparisons of wound size progression over the 14-day treatment period are presented in Table 1, which includes images from both the EF-treated and control groups, overlaid with a calibrated measurement grid for precise size estimation.

By Day 14, the EF-treated wounds exhibited near-complete closure with minimal scarring, averaging 0.1 cm in both length and width and a depth of 0.2 cm. In contrast, the control group retained larger wounds, averaging 0.40 cm in width, 0.60 cm in length, and 0.65 cm in diameter.

Specifically, by Day 7, the EF-treated wounds achieved an average closure rate of 82.0% ± 2.1%, significantly higher than the 70.75% ± 2.3% observed in the control group (*p* < 0.05). By Day 10, the closure rates reached 91.5% ± 1.4% for the EF group and 86.5% ± 1.8% for the control group. On Day 12, these rates further increased to 97.75% ± 1.2% and 91.5%± 1.5%, respectively. By the end of this study (Day 14), the EF-treated wounds achieved a near-complete closure of 99.75% ± 0.2%, while the control wounds reached 94.00% ± 1.7%, with all differences remaining statistically significant (*p* < 0.05).

Table 2 summarizes the wound area measurements and closure percentages across key time points in this study. These results indicate a significantly accelerated healing rate in the EF-treated wounds compared to the untreated control group.

**Figure 5:**
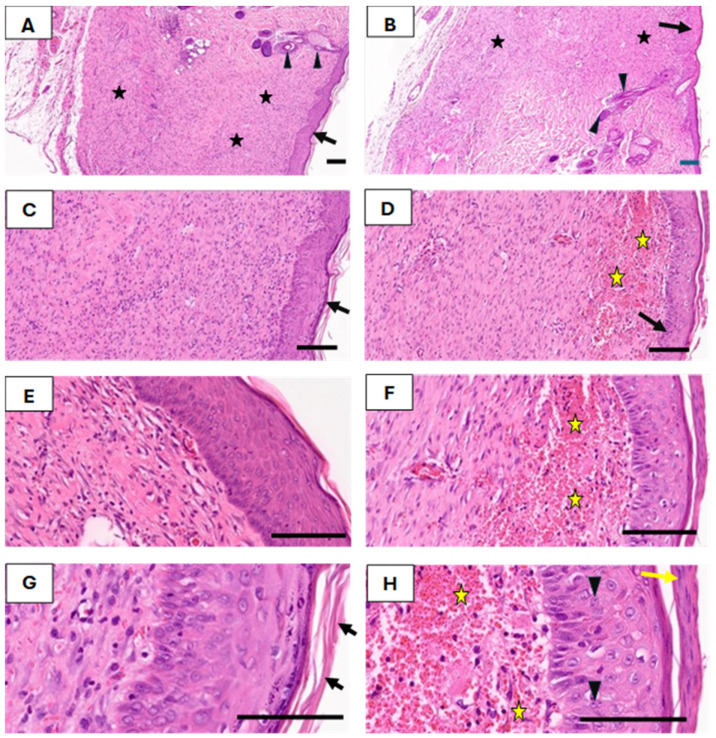
H&E-stained photomicrographs of healed skin from control (**A**,**C**,**E**,**G**) and EF-treated rats (**B**,**D**,**F**,**H**), highlighting differences in epidermal layers and subepithelial structures. Scale bars: 50 µm.

Histological examination revealed an increase in epidermal thickness in both groups, from an average of 31 µm in intact skin to 54 µm post-healing. A notable difference was observed in the stratum corneum; in the EF-treated group, this layer consisted of viable cells, whereas in the control group, it was composed of dead, cornified cells. Furthermore, the stratum spinosum in the EF group contained a significantly higher number of mitotically active cells, highlighting increased cellular proliferation. Histological sections stained with H&E (Figure 5) illustrate these distinctions. The healed skin in both groups showed intact epithelium with black arrows indicating specific points of interest. In the EF group, subepithelial blood vessels were more apparent (stars in panels D, F, and H), and the stratum corneum and spinosum displayed living cells and active cell division (yellow arrows and arrowheads in panel H), contrasting with the dead, cornified cells in the control group (arrows in panel G).

**Figure 6:**
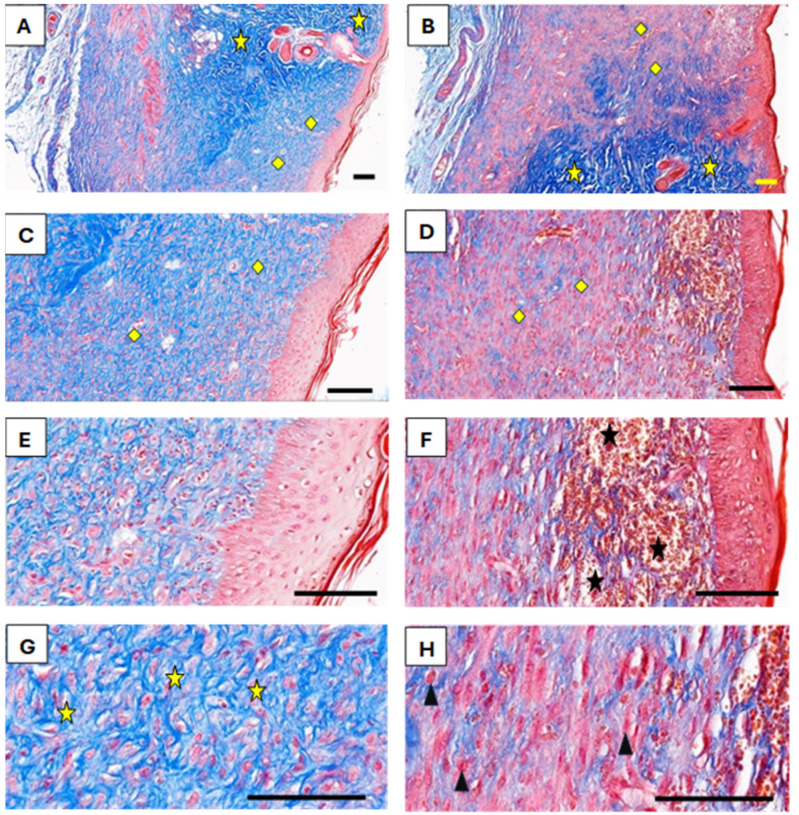
Photomicrographs comparing healed skin from control (**A**,**C**,**E**,**G**) and EF-treated rats (**B**,**D**,**F**,**H**), using Masson’s trichrome stain. Magnifications: (**A**,**B**) ×40; (**C**,**D**) ×100; (**E**,**F**) ×200; (**G**,**H**) ×400. Scale bars: 50 µm.

Another striking feature in EF-treated wounds was the presence of newly formed blood vessels in the subepithelial layer, as observed in Masson’s trichrome-stained sections (Figure 6). These vessels were more abundant in the EF group compared to the control group, indicating an angiogenic response triggered by EF exposure. Angiogenesis plays a critical role in delivering oxygen and nutrients to regenerating tissues, further supporting the beneficial effects of EF therapy.

A photomicrograph of the treated tissue revealed multiple cutaneous layers, including the epidermis (E), collagenous bundles (Cb), hair shaft (hs), hair follicle (hf), sebaceous gland (S), adipose tissue (Ad), blood vessels (bv), nerves (N), cutaneous muscle (MS), and subcutaneous tissue (sub). The images were captured at ×40 magnification, using an MS stain with a scale bar of 50 µm. Notably, in the photomicrographs, yellow stars indicate aggregated collagen bundles in intact skin, while yellow diamonds mark the absence of such bundles in healed wound areas. Black stars denote newly formed blood vessels in the subepithelial region, and black arrows highlight dense fibroblastic nuclei in the EF-treated dermis.

### 3.2. Connective Tissue Remodeling

The dermal layer structure also differed significantly between the two groups following healing. Neither the papillary nor reticular layers were distinguishable; instead, a homogeneous layer of collagenous fibers formed parallel to the epidermis. In EF-treated rats, this newly formed dermis was densely populated with fibroblastic nuclei (stained red), indicative of active extracellular matrix remodeling. In contrast, the control group predominantly showed collagenous fibers (stained blue) with fewer fibroblasts, suggesting a slower regenerative process. The Masson’s trichrome staining in Figure 6 highlights these differences. In both intact and healed skin, collagenous fibers in control and EF-treated rats did not form bundles in healed wounds. Additionally, numerous newly formed blood vessels were observed in the subepithelial area of EF-treated rats, further indicating enhanced healing.

### 3.3. Physiological Results

Physiological parameters, including heart rate (BPM), oxygen saturation (SpO2), and body temperature, were continuously monitored and recorded for both the experimental (EF-treated smart bandage) and control (non-EF smart bandage) groups. Automated sensor systems were used to ensure high-resolution data acquisition, with artifacts and signal drops removed to improve the reliability of the results.

Table 3 represent the mean values for each physiological parameter (BPM, SpO_2_, and temperature) for both Rat X (EF-treated) and Rat Control (No EF) across different days. The analysis revealed that rats in the EF-treated group maintained stable physiological parameters throughout this study, with no signs of tachycardia, which is often an early indicator of systemic infection or stress. The heart rate for both groups remained within the expected range for healthy Sprague–Dawley rats (~150–250 BPM), with no sustained elevation indicative of infection or inflammatory response.

To further analyze the physiological differences between the EF-treated (Rat X) and non-EF (Rat Control) groups, statistical comparisons were conducted for heart rate (BPM), oxygen saturation (SpO_2_), and body temperature over the study period. The mean values for each parameter were calculated across all recorded time points, and *t*-tests were performed to determine whether the observed differences were statistically significant.

The results indicate that the EF-treated group exhibited a significantly lower mean heart rate (211.5 BPM) compared to the control group heart rate (249.0 BPM) (*p* < 0.05), suggesting that electric field stimulation contributed to enhanced circulatory regulation and autonomic balance. SpO_2_ levels were in the EF group (around 96%) compared to the control group (92.6%) (*p* < 0.05). In contrast, temperature differences between the two groups were not statistically significant (*p* > 0.05), confirming that EF stimulation did not induce systemic fever or inflammatory responses (Table 4).

To assess the potential interdependence between heart rate (BPM), oxygen saturation (SpO_2_), and body temperature in the EF-treated group, Pearson correlation analyses were conducted. The results reveal a strong negative correlation between BPM and SpO_2_ (r = −0.912), suggesting that as heart rate decreases, oxygen saturation increases. This finding supports the hypothesis that EF stimulation optimizes oxygen utilization and circulatory efficiency, reducing cardiac workload while maintaining sufficient oxygen delivery.

A weak negative correlation was observed between BPM and temperature (r = −0.183), indicating that changes in heart rate were not strongly associated with thermoregulation, reinforcing the absence of a systemic stress response. Additionally, the correlation between SpO_2_ and temperature was slightly positive (r = 0.157), suggesting that minor fluctuations in oxygen levels may be linked to metabolic activity, though the effect was not statistically strong.

Overall, these correlations highlight the physiological stability of EF treatment, demonstrating that the smart bandage modulated cardiovascular responses without inducing adverse effects on thermoregulation or oxygen homeostasis. A summary of these correlations is provided in Table 5 below.

### 3.4. Predictive Modeling for Wound Healing Status

To further assess the effectiveness of physiological biomarkers in classifying wound healing outcomes, machine learning (ML) models were employed to predict wound condition as either “healed” or “not healed”. The predictive modeling utilized key biometric features recorded by the smart bandage, specifically healing duration, electric field exposure, BPM, SpO_2_, and temperature. The target variable was a binary classification of wound status, derived from wound closure percentage, with thresholds set at 80% closure or greater indicating “healed”.

A total of approximately 7000 data points were collected from 100 Sprague–Dawley rats over a 14-day period, with measurements recorded hourly for 5 h per day per animal. This high-resolution dataset provided a robust foundation for model training and evaluation, enabling time-series-based classification with a focus on daily wound progression.

Four supervised ML models were trained and tested: Logistic Regression, Random Forest, Support Vector Machine (SVM), and XGBoost. Model performance was evaluated using standard classification metrics including accuracy, precision, recall, F1 score, and area under the receiver operating characteristic curve (AUC). The results of all models are summarized in Table 6.

Among the tested models, XGBoost and Random Forest demonstrated exceptional performance, each achieving over 98% accuracy and AUC values of 99%, indicating strong discriminatory power in differentiating between healed and non-healed wounds. Logistic Regression also performed well, particularly in recall and AUC metrics, while SVM, although highly specific, exhibited limited sensitivity in identifying healed wounds.

These findings confirm that biometric data from the smart bandage—particularly heart rate, oxygen saturation, temperature, and electric field exposure timing—serve as powerful predictors of the wound healing trajectory. Notably, the XGBoost model emerged as the top-performing algorithm, making it a compelling choice for real-time integration into smart bandage systems for predictive wound management.

**Figure 7:**
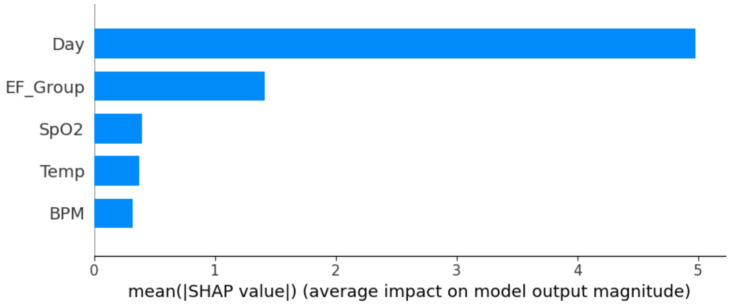
SHAP feature importance for predicting wound healing outcomes using XGBoost Model.

To further interpret the model’s decision-making, SHAP (SHapley Additive exPlanations) analysis was conducted. The results revealed for the XGBoost that healing duration and electric field exposure were the most influential features, followed by SpO_2_ and temperature, underscoring the critical roles of treatment duration and oxygen availability in successful wound resolution (Figure 7).

This intelligent classification pipeline underscores the transformative potential of combining biometric sensing with AI to enable early detection of delayed healing, reduce intervention time, and personalize treatment pathways, thereby advancing smart wound care toward real-time, predictive, and adaptive healthcare delivery.

## 4. Discussion

The spiral stainless steel electrodes used in our study play a pivotal role in enhancing the wound healing process through localized delivery of bioelectric cues. As highlighted in a review by Yu et al., conductive biomaterials can significantly influence the wound microenvironment by restoring the endogenous electric fields disrupted during injury [19]. These electric fields are known to promote key cellular processes such as keratinocyte migration, fibroblast proliferation, and angiogenesis, each crucial for efficient wound closure and tissue regeneration [21]. The spiral configuration of our electrodes offers an advantage by increasing the effective surface contact with the wound bed, enabling a more uniform distribution of electric stimulation. This design not only enhances electroactive modulation across the entire wound area but also maintains stable conductivity and mechanical integrity over time. These findings are consistent with the broader body of research on conductive wound dressings, affirming the potential of spiral stainless steel electrodes as a practical, scalable, and bioactive component for accelerating wound repair [19].

The findings of this study provide compelling evidence that electric field (EF)-enabled smart bandages significantly accelerate wound healing by promoting tissue regeneration and remodeling. By Day 14, the EF-treated group demonstrated near-complete wound closure (residual area: 0.01 cm^2^) compared to the control group (0.24 cm^2^). These results align with prior investigations that underscore the role of EF in modulating key wound healing processes. Notably, Zhao et al. [20] demonstrated that endogenous electric fields direct cell migration and regulate wound closure through PI3K and PTEN pathways, establishing a mechanistic foundation for EF therapy.

At Day 7, wounds in the EF-treated group achieved over 80% closure, qualifying as “healed” per study criteria, whereas the non-EF group reached only 70.75%. This finding supports previous studies showing that EF accelerates re-epithelialization and tissue granulation [21,22]. In a systematic review, Kloth [21] concluded that electrical stimulation significantly reduces healing time in various preclinical and clinical settings. Similarly, Rouabhia et al. [23] reported enhanced fibroblast proliferation and myofibroblast trans differentiation under EF exposure mechanisms that likely contributed to our observed healing dynamics.

Histological assessments further validated these effects. EF-treated wounds exhibited superior neovascularization, collagen alignment, and epithelial layer formation. These features are consistent with findings by Ud-Din and Bayat [24] and Ashrafi et al. [25], who observed that electrical stimulation promotes angiogenesis and extracellular matrix (ECM) remodeling, critical components of effective skin regeneration.

Our results are corroborated by Song et al. [26], who highlighted how electric cues influence the rate of wound healing and orientation of cellular processes. Moreover, increased ATP generation and protein synthesis under EF exposure demonstrated by Cheng et al. [27] may explain the enhanced metabolic and structural regeneration observed in our EF-treated group.

While the results of this study demonstrate the efficacy of the EF-enabled smart bandage in accelerating wound healing, it is important to note that standard clinical treatments such as silver dressings, hydrocolloids, or negative pressure wound therapy were not included as comparison groups. The decision to omit these was intentional, as the primary aim of this preclinical investigation was to isolate and evaluate the therapeutic contribution of electric field stimulation in a controlled setting. However, we recognize that comparisons with established standard-of-care treatments are essential to determine the true clinical value and translational potential of this technology. Future studies will incorporate such treatment arms to benchmark the smart bandage’s performance against widely accepted wound care modalities and better position it for clinical adoption.

Furthermore, although promising, these outcomes must be interpreted with caution due to the limitations of preclinical animal models. Variability in human wound etiology, comorbidities, and immune profiles could influence translational outcomes. Previous reviews, such as that by Nuccitelli [18], emphasized the complex interplay between endogenous electric fields and biological variability, urging more targeted large-animal and human trials.

The physiological monitoring aspect of our study revealed intriguing systemic effects. EF-treated animals exhibited a lower heart rate and marginally reduced SpO_2_ levels, potentially reflecting increased oxygen utilization at the wound site or altered autonomic regulation. Jia and Yang [29] highlighted the role of electric fields in influencing vascular dynamics and tissue oxygenation, supporting our interpretation.

Importantly, no significant differences in body temperature were noted between groups, suggesting that the EF-enabled smart bandage did not elicit systemic inflammation or thermoregulatory disturbances. These findings support the safety and tolerability of the intervention.

Although statistically significant differences were observed in heart rate (BPM) and oxygen saturation (SpO_2_) between the EF-treated and control groups, these variations are not considered physiologically significant. The average heart rate in the EF group was 211.5 BPM versus 249 BPM in the control group; both values fall within the normal range for Sprague–Dawley rats (typically 150–300 BPM) and do not indicate pathological bradycardia or tachycardia [27]. Similarly, SpO_2_ levels of 96% (EF group) and 92.6% (control group) are within acceptable physiological limits and do not suggest hypoxia or impaired respiratory function [27]. Importantly, no significant differences in body temperature were observed, indicating no signs of systemic inflammation or thermoregulatory disruption [27]. Therefore, while statistically detectable, these differences are unlikely to have had a substantial biological impact and instead support the overall safety and physiological stability of the smart bandage system throughout this study. This aligns with the design of the smart bandage, which operates under a low-voltage (2 V) and low-current regime specifically chosen to avoid heat generation. Although temperature was monitored systemically, future studies will aim to incorporate localized temperature and humidity sensors at the wound–tissue interface to better assess any microenvironmental changes induced by electrical stimulation and their potential effects on the wound healing trajectory.

In addition to physiological stability, histological evaluation provided further insights into the inflammatory response at the wound site. EF-treated wounds did not show evidence of excessive immune cell infiltration, edema, or tissue necrosis when compared to the control group, suggesting that the smart bandage did not provoke a heightened inflammatory reaction. This is consistent with previous findings demonstrating that moderate electric field stimulation can enhance tissue regeneration without triggering deleterious immune responses [23,24]. While the antibacterial properties of the bandage were not directly assessed in this study, the prior literature has reported that EF exposure may inhibit bacterial proliferation and biofilm formation, potentially contributing to infection control [17,29]. Future work will explore these antimicrobial effects more explicitly, as they may represent an added benefit of EF-enabled wound dressings in managing chronic or high-risk wounds.

In parallel with biological assessments, our predictive modeling component demonstrated the feasibility of machine learning (ML) for real-time wound monitoring. Using ~7000 biometric records, XGBoost and Random Forest models achieved over 98% accuracy in classifying healing outcomes. These results resonate with prior work by Berezo et al. [34], who successfully predicted chronic wound healing time using ML, and Jiang et al. [35], who developed a closed-loop smart bandage system integrating sensors and stimulators for wound care.

Our SHAP analysis revealed that healing duration and EF exposure were the most critical predictors across models—findings consistent with those of Wang and Meng [30], who emphasized the importance of stimulation duration in vascular remodeling. SpO_2_ and temperature were also key contributors, likely acting as surrogates for inflammation and perfusion, as also discussed in Hossain et al.’s smart bandage framework [36].

The integration of wearable sensors and biometric tracking into wound care aligns with recent trends in connected health technologies. Reviews by Shajari et al. [37] and Coman et al. [33] highlight the growing role of AI-integrated wearable systems in chronic disease and wound management areas where our study contributes new evidence.

However, our ML models, particularly SVM, faced limitations in generalizability and class imbalance. These issues are common in healthcare datasets and underscore the importance of model interpretability and validation. Logistic regression offered strong recall and transparency, supporting its use in clinical decision tools where explainability is vital.

Future work should focus on refining sensor design, enhancing model robustness, and expanding clinical datasets. Integrating wound imaging, molecular biomarkers, or tissue impedance measurements as suggested by Berry-Kilgour et al. [32] may further improve model precision.

## 5. Conclusions

This study set out to test the hypothesis that a smart bandage integrating spiral stainless steel electrodes for electric field (EF) therapy, combined with real-time physiological monitoring, can significantly accelerate wound healing and enable predictive assessment through machine learning. Our findings confirmed that EF-treated wounds demonstrated enhanced closure rates, superior tissue regeneration, and improved neovascularization compared to controls, with no systemic adverse effects. Additionally, biometric parameters such as heart rate, SpO_2_, and temperature captured continuously by the smart bandage were successfully used to predict healing outcomes with over 98% accuracy using XGBoost and Random Forest models.

This study introduced several key innovations: (1) a reusable, biocompatible spiral electrode design that ensures uniform EF distribution across the wound bed; (2) seamless integration of wearable sensors for closed-loop physiological monitoring; and (3) the application of interpretable AI models to forecast healing trajectories based on biometric inputs. These contributions collectively advance the field of intelligent wound care by bridging therapeutic stimulation with data-driven prognostics.

Looking ahead, future work will focus on scaling the technology for human use, refining sensor durability, and validating machine learning models in clinical settings. Additional efforts will explore integrating imaging biomarkers, biosignal analytics, and adaptive EF delivery to further personalize and optimize chronic wound treatment. This smart bandage platform paves the way for a new generation of responsive, AI-enabled wound management solutions.

## 6. Patents

This study is associated with a patent application for the smart bandage technology, which has been officially submitted for intellectual property rights protection. The application was filed under the title “Smart Bandage” with the Saudi Authority for Intellectual Property (SAIP) under application number SA 1020242555 on 13 May 2024.

## Figures and Tables

**Table 1:** Wound images from Days 0, 7, 10, 12, and 14 in both EF-treated and control groups, illustrating wound size reduction over time. Each image is overlaid with a measurement grid, where each square represents 0.1 cm × 0.1 cm, enabling direct visual estimation and comparison of wound dimensions between groups.

Date	Rat Control	Rat X-EF
Day 0	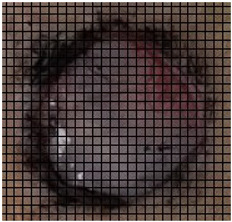	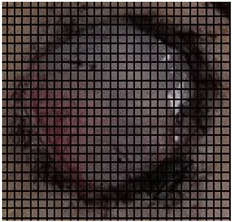
Day 7	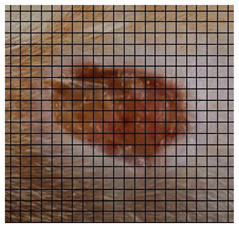	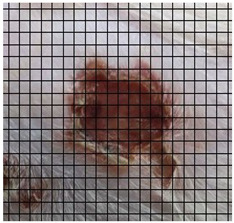
Day 10	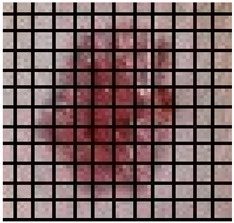	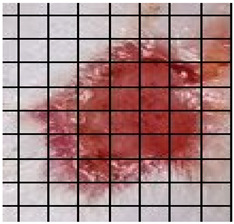
Day 12	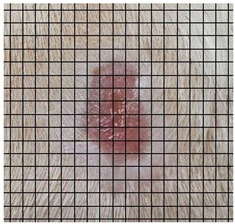	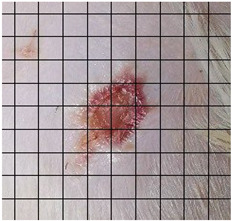
Day 14	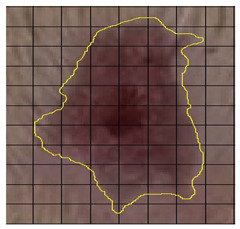	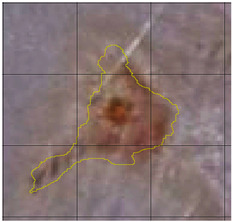

**Table 2:** Comparative wound healing efficiency in EF-treated and control groups.

Day	Wound Area (cm^2^)—Rat Control	Wound Closure (%)—Rat Control	Wound Area (cm^2^)—Rat X (EF)	Wound Closure (%)—Rat X (EF)	*t*-Test (*p*-Value)	
					Wound Area	Wound Closure
1	4	0.00%	4	0.00%	>0.05	>0.05
7	1.17± 0.15	70.75% ± 2.3%	0.72± 0.08	82% ± 2.1%	<0.05	<0.05
10	0.54 ± 0.05	86.5% ± 1.8%	0.30 ± 0.03	91.5% ± 1.4%	<0.05	<0.05
12	0.34 ± 0.04	91.5%± 1.5%	0.09± 0.01	97.75% ± 1.2%	<0.05	<0.05
14	0.24 ± 0.03	94.00% ± 1.7%	0.01± 0.005	99.75% ± 0.2%	<0.05	<0.05

**Table 3:** Physiological parameter comparison between EF-treated and control groups for selected days.

Day	BPM (Rat X—EF)	BPM (Rat Control)	SpO_2_ (Rat X—EF)	SpO_2_ (Rat Control)	Temp (Rat X—EF)	Temp (Rat Control)
	Mean± SD					
0	240 (±10)	250 (±10)	95 (±2)	94 (±2)	39 (±0.5)	38.5 (0.5)
6	200 (±5)	225 (±5)	96 (±1)	94 (±1)	39.5 (±0.2)	38.5 (±0.2)
8	210 (±5)	240 (±6)	97 (±1)	95 (±1)	40.2 (±0.3)	38.8 (±0.2)
9	205 (±5)	230 (±5)	98 (±1)	95 (±1)	41.0 (±0.4)	39.0 (±0.3)
10	215 (±5)	235 (±6)	97 (±1)	96 (±1)	39.8 (±0.3)	38.7 (±0.2)
12	190 (±5)	220 (±5)	95 (±1)	92 (±1)	39.2 (±0.2)	38.4 (±0.2)

**Table 4:** Statistical comparison of physiological parameters between EF-treated and non-EF rats.

Parameter	Rat X Mean (EF)	Rat Control Mean (Non-EF)	*t*-Test (*p*-Value)
Heart Rate (BPM)	211.5	249	<0.05
SpO_2_ (%)	96	92.6	<0.05
Temperature (°C)	39	38.53	>0.05

**Table 5:** Pearson correlation analysis of physiological parameters in EF-treated rats.

Metric Pair	Pearson Correlation Coefficient
BPM–Temperature	−0.183 (weak negative correlation)
BPM–SpO_2_	−0.912 (strong negative correlation)
SpO_2_–Temperature	0.157 (weak positive correlation)

**Table 6:** Performance metrics of supervised machine learning models for wound healing classification.

Model	Accuracy	Precision	Recall	F1 Score	AUC
Logistic Regression	0.91	0.78	0.82	0.80	0.93
Random Forest	0.98	0.95	0.96	0.96	0.99
SVM	0.79	0.97	0.06	0.11	0.93
XGBoost	0.98	0.94	0.96	0.95	0.99

## Data Availability

Data supporting the results reported in this study are available upon reasonable request. The dataset generated and analyzed during this study is not publicly available. Requests for access to the data can be directed to the corresponding author and will be evaluated in accordance with institutional and ethical guidelines.
